# Expression of a disintegrin and metalloprotease (ADAM and ADAMTS) enzymes in human non-small-cell lung carcinomas (NSCLC)

**DOI:** 10.1038/sj.bjc.6602990

**Published:** 2006-02-21

**Authors:** N Rocks, G Paulissen, F Quesada Calvo, M Polette, M Gueders, C Munaut, J-M Foidart, A Noel, P Birembaut, D Cataldo

**Affiliations:** 1 Laboratory of Pneumology and Laboratory of Tumor and Development Biology, Center for Biomedical Integrative Genoproteomics (CBIG), University of Liège and Centre Hospitalier Universitaire de Liège (CHU-Liège), Avenue de l’Hôpital, CHU, Sart-Tilman, Liège 4000, Belgium; 2 INSERM U514, Laboratory Pol Bouin, Hôpital Maison Blanche CHU, Reims, France

**Keywords:** ADAM, ADAMTS, lung, proteinases, VEGF

## Abstract

A Disintegrin and Metalloprotease (ADAM) are transmembrane proteases displaying multiple functions. ADAM with ThromboSpondin-like motifs (ADAMTS) are secreted proteases characterised by thrombospondin (TS) motifs in their C-terminal domain. The aim of this work was to evaluate the expression pattern of ADAMs and ADAMTS in non small cell lung carcinomas (NSCLC) and to investigate the possible correlation between their expression and cancer progression. Reverse transcriptase–polymerase chain reaction (RT–PCR), Western blot and immunohistochemical analyses were performed on NSCLC samples and corresponding nondiseased tissue fragments. Among the ADAMs evaluated (ADAM-8, -9, -10, -12, -15, -17, ADAMTS-1, TS-2 and TS-12), a modulation of ADAM-12 and ADAMTS-1 mRNA expression was observed. Amounts of ADAM-12 mRNA transcripts were increased in tumour tissues as compared to the corresponding controls. In sharp contrast, ADAMTS-1 mRNA levels were significantly lower in tumour tissues when compared to corresponding nondiseased lung. These results were corroborated at the protein level by Western blot and immunohistochemistry. A positive correlation was observed between the mRNA levels of ADAM-12 and those of two vascular endothelial growth factor (VEGF)-A isoforms (VEGF-A_165_ and VEGF-A_121_). Taken together, these results providing evidence for an overexpression of ADAM-12 and a lower expression of ADAMTS-1 in non-small-cell lung cancer suggest that these proteases play different functions in cancer progression.

Lung cancer is one of the most common causes of cancer death in Europe and in the United States and disease frequency rapidly increased over the last decades. Accumulating evidence demonstrated the important role of proteolytic enzymes such as matrix metalloproteinases (MMPs) in cancer progression (Egeblad and Werb, 2002; Overall and Lopez-Otin, 2002; Handsley and Edwards, 2005). In contrast, to date, no information is available on the putative relationship existing between lung cancers and MMP-related enzymes such as A Disintegrin and Metalloprotease (ADAM) and ADAM with ThromboSpondin-like motifs (ADAMTS) proteases.

A disintegrin and metalloprotease is a family of transmembrane proteases displaying multiple functions among which 21 human ADAM genes likely encode active proteases (Puente *et al*, 2003). A disintegrin and metalloprotease with thrombospondin (TS)-like motifs are secreted molecules bearing TS motifs in their C-terminal domain (Killar *et al*, 1999; Kaushal and Shah, 2000; Tang, 2001; Cal *et al*, 2002). On the basis of their structure, ADAMs and ADAMTS are thought to mediate a wide variety of activities including proteolysis, adhesion, cell fusion and signalling (Porter *et al*, 2005).

Although their functions are not yet fully elucidated, an upregulation of ADAMs and ADAMTS has been observed in some pathological conditions. A recent report suggests that ADAM-8 is overexpressed by lung cancer cells and is detectable in patient's serum (Ishikawa *et al*, 2004). A disintegrin and metalloprotease-10 is upregulated in some tumour cells (Wu *et al*, 1997) and in arthritic chondrocytes (McKie *et al*, 1997). A disintegrin and metalloprotease-12 and 15 are also abundantly expressed in cells derived from haematological malignancies (Wu *et al*, 1997). Monocytes from lung cancer patients produce elevated levels of mature TNF-*α* (Trejo *et al*, 2001) which could result from the shedding of pro-TNF-*α* by ADAM-17 (TACE) (Black *et al*, 1997). ADAM with TS-like motifs-1 (ADAMTS-1) is expressed in colon cancer cells (Kuno *et al*, 1997).

Even if the implication of ADAMs and ADAMTS during lung cancer progression remains unclear, it is conceivable that, as shown for MMPs, these related proteases contribute to extracellular matrix degradation, cell–cell adhesion, cell proliferation, cell migration as well as to the processing of cytokines or growth factors (Egeblad and Werb, 2002; Overall and Lopez-Otin, 2002; Handsley and Edwards, 2005). The putative interest of studying ADAMs and ADAMTS in lung carcinomas is reinforced by a recent report of Lemjabbar *et al* (2003), demonstrating that tobacco smoke-induced bronchial epithelial cell proliferation is mediated by the cleavage of amphiregulin, a ligand of EGF receptor, by ADAM-17. A disintegrin and metalloprotease-15 (ADAM-15) deficiency in mice is associated with impaired pathological angiogenesis and reduced tumour growth (Horiuchi *et al*, 2003). In addition, recently, an interplay between vascular endothelial growth factor (VEGF) and metalloproteases has been reported. This new concept is supported by (1) the ability of ADAMTS-1 to bind VEGF and functionally inactivate VEGFR2 (Iruela-Arispe *et al*, 2003), (2) the existence of a correlation between VEGF and some MMP expression in tumours, (3) the upregulation of VEGF-A expression by the active form of membrane type-1 MMP (MT1-MMP, MMP14) (Munaut *et al*, 2003), (4) the reduction of VEGF expression in tumour cells by physiological inhibitor (TIMP-2) (Hajitou *et al*, 2001) or synthetic inhibitor (Sounni *et al*, 2004) of MMPs. These observations suggest the involvement of ADAM, ADAMTS and MMP members in the control of angiogenesis, a key step of metastatic dissemination.

The aim of the present work was to determine the expression profile of selected ADAMs and ADAMTS in NSCLC as well as to study the putative correlation existing between these proteases and VEGF isoform expression levels and the disease stage.

## MATERIALS AND METHODS

### Tumour tissue samples

Surgical samples from non-small-cell lung tumours and corresponding lung control tissues were obtained from 39 patients with squamous cell lung cancers or adenocarcinomas. Characteristics of patients and histological subtypes are described in Table 1. The protocol of the study was approved by the Ethical Committee of the Hôpital Maison Blanche, Reims and informed consent was obtained from all patients before surgery.

### Cell culture and RNA isolation

*Lung cancer cell lines*: BEAS-2B were purchased from ATCC, while BZR, BZR-T33, and 16-HBE were kindly provided by Dr CC Harris (National Institute of Health, Bethesda, MD, USA). All cell lines were cultured in Dulbecco's modified Eagle's medium (DMEM, Invitrogen, Merelbeke, Belgium) supplemented with 10% foetal bovine serum, 5% penicillin–streptomycin (Invitrogen, Merelbeke, Belgium) and 5% glutamine in 5% CO_2_ at 37°C. BEAS-2B cells were cultured in collagen-coated Petri dishes in Airway Epithelial Cell Basal Medium (Promocell, Heidelberg, Germany).

Total RNAs were extracted from normal and tumoral lung tissues as well as from 16-HBE, BZR, BZR-T33 and BEAS-2B cells by the use of the RNA Easy Qiagen Kit (Qiagen, MD, USA). Total RNA concentrations were measured using the RiboGreen RNA quantification Kit (Molecular Probes, OR, USA). Samples were stored at −80°C.

### Design of oligonucleotide primers

The design of oligonucleotide primers specific for the different targets was based on sequences available in the Genbank. Primers, obtained from Eurogentec (Seraing, Belgium), were designed to anneal to distinct exons and the specificity of the selected sequences was verified with the NCBI BLASTN program (Table 2). Polymerase chain reaction products obtained with each pair of primers were digested with appropriate restriction enzymes to verify the specificity of amplification.

### Semiquantitative RT–PCR

The mRNA expression levels of ADAMs, ADAMTS and VEGF-A were determined by semiquantitative RT–PCR. Reverse transcriptase–polymerase chain reaction was performed on 10 ng of total RNA at 70°C during 15 min using the GenAmp thermostable RNA RT–PCR Kit (Applied Biosystems, Foster City, CA, USA). Reverse transcriptase–polymerase chain reaction conditions and primers used to measure VEGF-A expression were those previously described (Hajitou *et al*, 2001). The intensity of each band was measured with the Quantity One software (Biorad, Hercules, USA). To normalise mRNA levels in different samples, the value of the band corresponding to each mRNA level was divided by the intensity of the corresponding 28S rRNA band used as an internal standard.

### Real-time PCR

Total RNA (500 ng) was reverse transcribed in a 15 *μ*l reaction using 50 ng of random hexamers and ThermoScript reverse transcriptase (Life Technologies, Paisley, UK) according to the manufacturer's instructions. Primers for ADAM-12 and ADAMTS-1 as well as the corresponding TaqMan probe were designed using PRIMER EXPRESS 1.0 software (Applied Biosystems, Foster City, CA, USA). In order to avoid genomic DNA amplification, primers were chosen within different exons, close to intron–exon boundaries. The 18S ribosomal RNA gene was used as an endogenous control to normalise RNA amounts in each sample. TaqMan 18S ribosomal primers as well as the VIC-labelled probe were used according to the manufacturer's instructions (Applied Biosystems, Foster City, CA, USA). Polymerase chain reaction (PCR) reactions were performed on a 96-well optical plate with the Platinum Super Mix (Invitrogen, Paisley, UK) using 5 *μ*l of diluted cDNA (equivalent to 10 ng total RNA), 200 nM of the probe and 400 nM primers in a 25 *μ*l final reaction mixture. Each of the 40 PCR cycles consisted of 15 s of denaturation at 95°C and hybridisation of probes and primers for 1 min at 60°C. Real-time quantitative PCR analyses for ADAM-12 and ADAMTS-1 were performed using the ABI PRISM 7700 Sequence Detection System instrument and software (Applied Biosystems, Foster City, CA, USA). The amount of target gene was divided by the 18S rRNA amount to obtain a normalised target value. Each experiment was performed in duplicate and the Standard Error of mean (s.e.m) has been calculated on the basis of the two experiments.

### Western blot analysis

Proteins were isolated from tumour and control tissues by urea extraction (Cataldo *et al*, 2002). Samples were migrated on a 12% polyacrylamide gel and transferred to a PVDF membrane (Perkin Elmer Life Sciences Inc., Boston, MA, USA). In order to normalise Western blots data, tissue extracts corresponding to 20 *μ*g of total proteins were loaded for each patient. Anti-ADAM-12 (1/100) or anti-ADAMTS-1 (1/500) antibody (Santa Cruz Biotechnologies Inc., Sigma-Aldrich, Belgium) was applied overnight. Proteins were finally detected by chemiluminescence with rabbit anti-goat-IgG (DAKO, Glostrup, Denmark) diluted 1/1000 coupled with HRP immunoreactives.

### Immunohistochemistry

Tissue sections were incubated for 1 h with polyclonal antibodies recognising either ADAM-12 (Sigma, St Louis, MI, USA) or ADAMTS-1 (Santa Cruz Biotechnologies Inc., Santa Cruz, CA, USA) protein and after rinsing were incubated for 30 min with anti-rabbit antibodies coupled to horseradish peroxidase-labelled dextran polymers (Envision, DAKO, Glostrup, Denmark) for ADAM-12 or with rabbit anti-goat IgG antibodies (DAKO, Glostrup, Denmark) for ADAMTS-1. Slides were finally incubated with 3-amino-9-ethylcarbazol (AEC) (DAKO, Glostrup, Denmark) and the sections were counterstained with haematoxylin.

### Statistical analysis

Data are reported as mean±s.e.m. and statistical analysis was performed by the Mann–Whitney test. Correlations were measured by the Spearman's test. The threshold for significance was set at *P*<0.05.

## RESULTS

### Semiquantitative RT–PCR and real-time PCR

**Figure 1:**
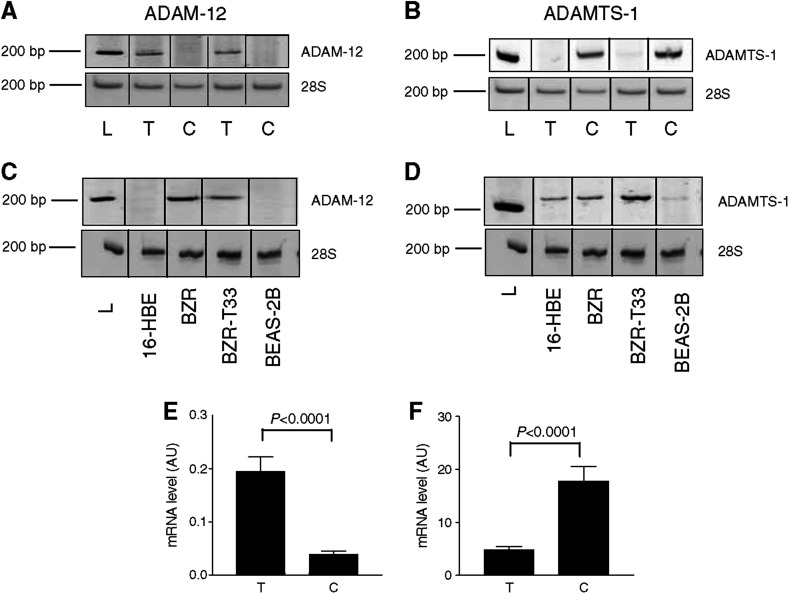
RT–PCR analysis of ADAM-12 and ADAMTS-1 in human tumour samples and normal lung tissues (*n*=39) and in lung cancer cell lines. (**A**) mRNA transcripts of total ADAM-12. The expression of ADAM-12 mRNA is significantly higher in tumours (T) than in their control tissues (C). (**B**) Expression of ADAMTS-1 mRNA. ADAMTS-1 expression is lower in tumour tissues when compared to the corresponding control tissue. L: 200 bp molecular weight (Smart Ladder, Eurogentec, Seraing, Belgium). (**C**–**D**) ADAM-12 (left panel) and ADAMTS-1 (right panel) mRNA expression in human lung cancer cell lines. The bottom line represents the 28S RNA. (**E**–**F**) Results of quantitative real-time PCR for ADAM-12 and ADAMTS-1, expressed as arbitrary units (AU) normalised to the 18S rRNA (described in the Materials and Methods section).

Polymerase chain reaction analyses were performed on NSCLC obtained by surgery from 34 men and five women. Table 1 summarises the characteristics of patients, their TNM states based on pathological examination (pTNM) and histological subtype of tumours. The mRNA expression levels were determined on each human tumour sample and their corresponding control lung tissue by semiquantitative RT–PCR (Table 3). Among ADAMs and ADAMTS evaluated, a difference in ADAM-12 and ADAMTS-1 mRNA levels was evidenced between cancer and control samples. In contrast, no modulation of ADAM-8, -9, -10, -15, -17 and ADAMTS-2 and -12 was observed in the two sample groups (Table 3). Interestingly, the amounts of ADAM-12 transcripts normalised to 28S rRNA were significantly higher in lung tumours when compared to the matched normal tissues (*P*=0.0005) (Figure 1A). Inversely, ADAMTS-1 mRNA was expressed at lower levels in tumour samples than in normal lung tissues (*P*<0.0001) (Figure 1B). To confirm these results, quantitative real-time PCR was then performed. Amounts of ADAM-12 transcripts were confirmed to be significantly increased in tumours (0.2±0.03 in tumours *vs* 0.04±0.006 in normal tissues; *P*<0.0001) (Figure 1E). Again, amounts of ADAMTS-1 mRNA copies were found to be lower in tumours than in control samples (4.9±0.57 in tumours *vs* 17.8±2.7 in controls; *P*<0.0001) (Figure 1F).

**Figure 2:**
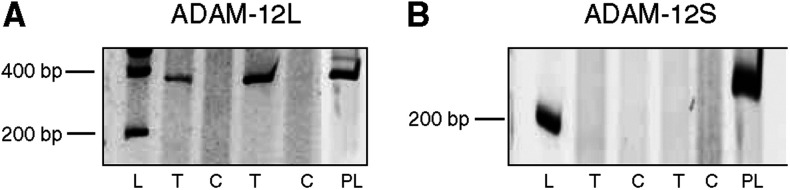
RT–PCR analysis of spliced variants of ADAM-12. Long membrane-anchored variant (ADAM-12L) and short secreted variant (ADAM-12S) in tumour and normal lung tissue (*n*=39). (**A**) mRNA expression of ADAM-12L. The expected molecular weight was 340 bp for ADAM-12L. (**B**) mRNA expression of ADAM-12S. The expected molecular weight was 220 bp for the short form. L: 200 bp molecular weight, T: tumoral tissue, (**C**): corresponding control tissue. PL: human placental mRNA used as control.

Since two forms of ADAM-12 resulting from an alternative splicing have been described, two additional pairs of primers have been designed near to the splicing region to determine the relative amounts of these isoforms. ADAM-12L (membrane-bound long variant) transcripts were overexpressed in tumours when compared to controls, while no expression of short form of ADAM-12 (ADAM-12S) (secreted short variant) was detected in the lung tissues examined (Figure 2). This result demonstrates that almost the vast majority of ADAM-12 expressed in tumour tissue corresponds to the membrane-bound form of ADAM-12.

No significant differences were found regarding expression levels of ADAM-12 and ADAMTS-1 when considering the different TNM states or survival (data not shown). However, in the adenocarcinoma subgroup, we showed an increase of ADAM-12 mRNA in the N0 stages when compared to N1 or N2 stages. There were no significant differences for the expression of any ADAM or ADAMTS protease between the squamous cell and adenocarcinoma groups.

We next analysed ADAM expression in human epithelial lung cell lines (16-HBE, BZR, BZR-T33 and BEAS-2B cells). Almost all cell lines tested expressed ADAM-9, -10, -12, -15 and ADAM-17 (data not shown). A disintegrin and metalloprotease-12 mRNA were not detected in immortalised BEAS-2B and 16-HBE cells derived from normal epithelial cells (Figure 1C). In sharp contrast, ADAM-12 was strongly expressed in two cell lines, BZR and BZR-T33, derived from BEAS-2B cells by infection with recombinant retrovirus Zip-neo-v-Ha-ras or derived from a tumour formed by BZR cells injected subcutaneously into nude mouse, respectively. These two cell lines expressing ADAM-12 (BZR and BZR-T33) have been reported to display a more invasive behaviour when implanted in mice (Bonfil *et al*, 1989) than cells not expressing ADAM-12 (BEAS-2B; 16-HBE). ADAM with TS-like motifs-1 (ADAMTS-1) mRNA expression was also investigated in these cell lines (Figure 1D).

### Western blot analysis

**Figure 3:**
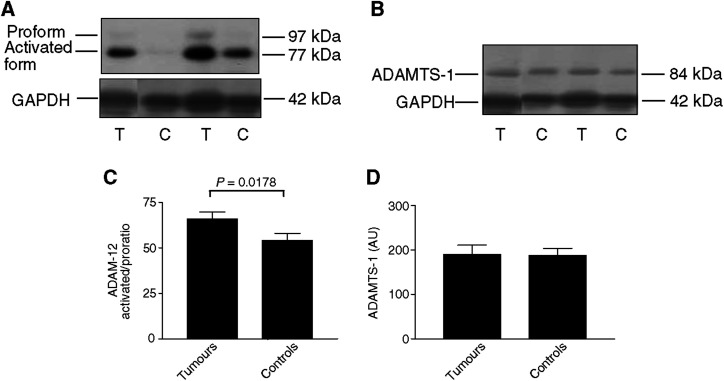
Western blotting of ADAM-12 protein immunoreactivity (**A**) and ADAMTS-1 (**B**) in normal and tumour lung tissues (*n*=39). For each sample, the lower panel corresponds to the house keeping gene product glyceraldehyde-3-phosphate dehydrogenase (GAPDH) used as a loading control. T: tumour tissue, C: control tissue. ADAM-12 activated/proratio (densitometric analysis of the immunoreactivity of the activated form of ADAM-12 (77 kDa) normalised by the immunoreactivity of the proform (97 KDa)) (**C**) and levels of immunoreactivity corresponding to activated ADAMTS-1 (84 kDa) (**D**).

The activation status of ADAM-12 and ADAMTS-1 in tumours and corresponding control tissues was investigated by Western blotting. The 97 kDa proform and the 77 kDa activated form of ADAM-12L were detectable in samples (Figure 3A). The results confirm at the protein level the significant increase in tumour samples *vs* control samples observed at the mRNA level. Moreover, the ratio between activated and proforms was higher in tumours than in corresponding controls (65.8±3.85 *vs* 54±3.91) (Figure 3C).

No difference in ADAMTS-1 production was observed between tumour and control samples (Figure 3B and D).

### VEGF-A semiquantitative RT–PCR

**Figure 4:**
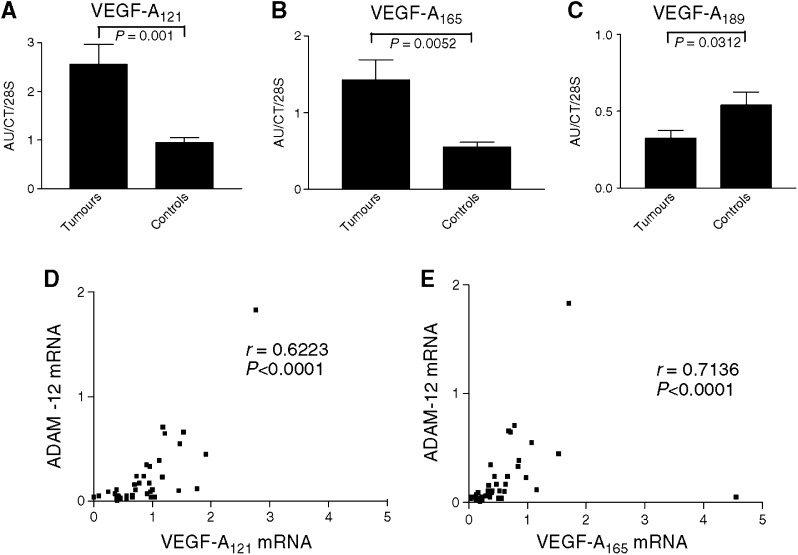
VEGF-A_121_ and VEGF-A_165_ mRNA levels are more elevated in tumour samples (T) (*P*<0.05) (**A**–**B**) while VEGF-A_189_ mRNA levels are lower in the squamous carcinomas and adenocarcinomas (*P*<0.001) (**C**). Results are expressed as arbitrary units (AU) divided by the values of an internal control and are normalised for the amount of 28S rRNA. In tumour samples, a positive correlation between ADAM-12 and VEGF-A_121_ (**D**) and VEGF_165_ (**E**) mRNA levels has been observed (*P*<0.0001).

The analysis of VEGF-A mRNA expression revealed an overexpression of VEGF-A_121_ and VEGF-A_165_ (Figure 4A and B) and lower levels of VEGF-A_189_ mRNA (Figure 4C) in all tumour samples when compared to corresponding control lungs. A positive correlation was observed between the mRNA levels of ADAM-12 and those of VEGF-A_121_ (*P*<0.0001) or VEGF-A_165_ (*P*<0.0001) in tumour samples (Figure 4D and E). Vascular endothelial growth factor-A_121_ expression was also significantly increased in tumours displaying N2 status as compared to those without nodal involvement (N0).

### Immunohistochemistry

**Figure 5:**
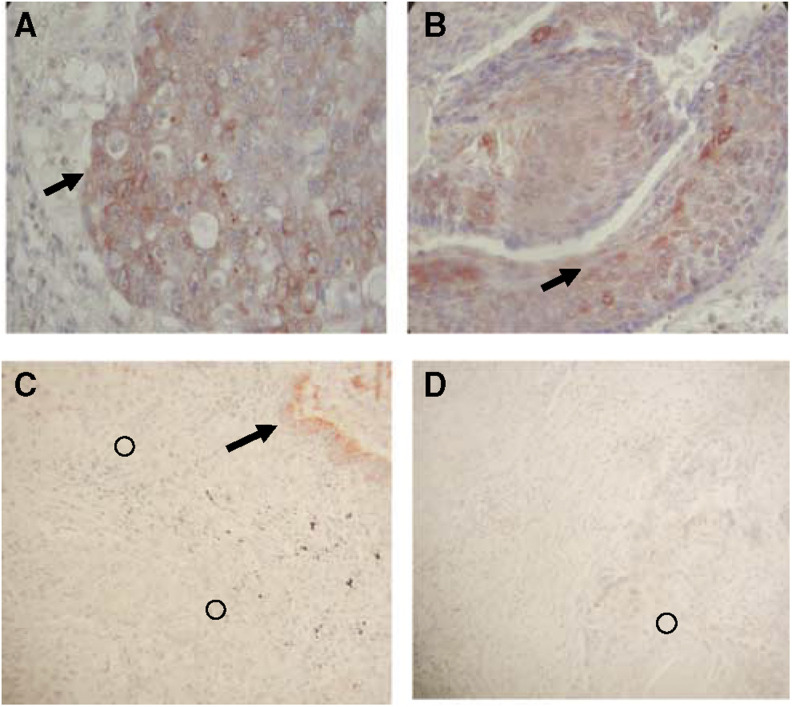
Immunohistochemistry. Paraffin sections of adenocarcinoma (**A**) and squamous cell lung cancer (**B**) were subjected to ADAM-12 immunohistochemistry as reported in the Materials and Methods section. Tumour cells were strongly labelled (arrows) by anti-ADAM-12 antibody. Paraffin sections of adenocarcinoma (**C**) and squamous cell lung cancer (**D**) were subjected to ADAMTS-1 immunohistochemistry as reported in the Materials and Methods section. Immunostaining for ADAMTS-1 was localised at the level of normal bronchi surrounding the tumour (arrow) in NSCLC lung fragments while tumour cells (circles) did not display any staining with ADAMTS-1 antibody.

A disintegrin and metalloprotease-12 immunoreactivity was mainly detected in tumour cells (Figure 5A and B). Some immunostaining was also observed, as expected, in normal smooth muscle surrounding the tumour. In normal lung, some inflammatory cells were positively stained while epithelial cells were always negative.

ADAMTS-1 immunoreactivity was mostly seen in normal epithelial bronchial cells in control lung tissues as well as in normal bronchi surrounding tumour nodules in diseased lung (Figure 5C and D). The transition of normal epithelial cells into tumour cells was associated with a loss of ADAMTS-1 immunostaining.

## DISCUSSION

The present study was designed to investigate the potential involvement of ADAMs and ADAMTS in the pathogenesis of lung carcinomas. Indeed, ADAMs and ADAMTS belong to the adamalysin family, related to snake venom proteases, among which many members are implicated in different loops of reciprocal interactions with some mediators of inflammation such as TNF-*α* (Rosendahl *et al*, 1997; Moss *et al*, 2001) and growth factors including TGF-*α* and -*β* (Hinkle *et al*, 2003; Le Pabic *et al*, 2003).

We describe for the first time an increased production of ADAM-12 both at the mRNA and protein levels in human lung squamous cell carcinomas and adenocarcinomas. On the opposite, we report a decreased expression of ADAMTS-1 mRNA in squamous cell carcinomas and adenocarcinomas when compared to corresponding control samples. In addition, this study provides the first evidence for a link between ADAM-12 expression and VEGF-A_121_ or VEGF-A_165_ expression. An overproduction of ADAM-12 in lung tumours has been evidenced by RT–PCR, real-time and Western blot analysis. As an alternative splicing has been reported for ADAM-12 and as the secreted short form of ADAM-12 (ADAM-12S) was described to be expressed by tumours such as rhabdomyosarcoma and HU-1 cells (Gilpin *et al*, 1998), we assessed our tumour samples for the expression of ADAM-12L and ADAM-12S by specific RT–PCR. Only ADAM-12L was detectable in lung cancer samples, suggesting that ADAM-12 was mainly cell membrane associated. Accordingly, Western blot analyses confirmed the presence of both pro and activated forms of ADAM-12L but not of ADAM-12S.

A disintegrin and metalloprotease-12 overexpression in tumour samples could be related either to an increased expression by carcinoma cells themselves or by some stromal cells such as myofibroblasts surrounding tumour islets. We demonstrate in the present paper that cultured BZR lung-derived cancer cell lines expressed ADAM-12 mRNA, while 16-HBE and BEAS-2B cells derived from normal epithelial cells did not express significant levels of ADAM-12 mRNA. Others have reported an overexpression of ADAM-12 in liver carcinomas (Le Pabic *et al*, 2003), and its levels in urine from patients have been correlated with survival in breast cancer (Roy *et al*, 2004). These data, taken together with the faint expression of ADAM-12 mRNA levels in healthy lung extracts, indicate that tumour cells are probably the main producer of ADAM-12 in our experimental conditions. In accordance, immunohistochemical analysis revealed ADAM-12 production by tumour cells. The fact that invasive cell lines (BZR and BZRT33) expressed huge amounts of ADAM-12 when compared to cell lines derived from normal epithelium (BEAS-2B and 16HBE) suggests that ADAM-12 may play a role in the cascade of events leading to the invasive phenotype. As demonstrated recently, ADAM-12 could play an important role in cell adhesion (Zolkiewska, 1999; Iba *et al*, 2000) and, therefore, its increased expression in lung cancer cells could be mandatory for tumour cell migration and invasion through a control of cell–matrix interactions. In addition, ADAM-12 could be of particular importance in the processes leading to cell proliferation since it sheds the soluble heparin-binding epidermal growth factor (Asakura *et al*, 2002). Furthermore, the correlation observed between ADAM-12 and VEGF transcripts is of great interest since angiogenesis is an essential step of tumour progression. The finding of a positive correlation between ADAM-12 and VEGF-A_121_ and VEGF-A_165_ isoforms, which are proangiogenic, in all tumour samples reinforce the hypothesis of a specific role for ADAM-12 in tumour-associated angiogenic process. The potential effect of ADAM-12 on angiogenesis could occur either directly by activating some mediators implicated in angiogenesis as demonstrated for some MMPs or by inactivating angiogenesis inhibitors (Munaut *et al*, 2003; Maquoi *et al*, 2004; Noel *et al*, 2004). Alternatively and as suggested for other proteases, ADAMs could release proangiogenic factors trapped in the extracellular matrix by degrading its components (Werb *et al*, 1999). Nevertheless, our results are only correlative regarding the relationship between ADAM-12 and VEGF isoforms expression and further studies are needed to confirm our results and to precisely dissect the potential mechanisms linking ADAM proteases and tumour angiogenesis. The increased levels of VEGF-A found in tumours associated with N2 states when compared to those associated with N0 is in line with a recent report (Iwasaki *et al*, 2004) showing that VEGF expression in tumour samples is correlated with markedly poor prognosis.

ADAMTS-1 is a secreted protein, which can bind matrix through interaction between its TS-1 motifs and heparin sulphate. It plays significant roles in organogenesis, inhibition of VEGF and fibroblast growth factor (FGF-2)-induced angiogenesis (Iruela-Arispe *et al*, 2003) and is associated with IL-1 and LPS-induced inflammation (Kuno *et al*, 1997). ADAMTS-1 has been shown to bind VEGF-A_165_ and to inhibit VEGF-A_165_-stimulated VEGF-R2 phosphorylation (Luque *et al*, 2003). In this context, our finding of a significant decrease of ADAMTS-1 in lung cancer is of particular importance. To the best of our knowledge, the present study is the first report of a negative association between a member of ADAMTS-1 and lung cancers. Dunn *et al* (2004) have previously reported such a negative association for ADAMTS-8 which was in that case associated with a promoter hypermethylation in cancers. A negative association has been demonstrated in pancreatic cancers (Masui *et al*, 2001) and breast cancers (Porter *et al*, 2004). The potential implication of ADAMTS-1 in regulation of cancer-related angiogenesis should be studied more in depth especially regarding factors stimulating or inhibiting this protease. Unveiling such factors could be particularly relevant in the setting of new therapeutic options acting through angiogenesis modulation.

In conclusion, we demonstrate in the present study that members of the ADAM and ADAMTS subfamilies are differently modulated in lung cancers suggesting different functions for individual ADAM(TS) in the development, and progression of lung carcinomas.

## Figures and Tables

**Table 1:** Characteristics of tumours and patients

	**Adenocarcinoma**	**Squamous cell**
Number of samples	26	13
Mean age	60	67
Sex ratio (M/F)	22/4	12/1
T	T_1_: 2	T_1_: 2
	T_2_: 20	T_2_: 9
	T_3_: 4	T_3_: 2
		
N	N_0_: 15	N_0_: 6
	N_1_: 5	N_1_: 7
	N_2_: 5	
	N_3_: 1	
		
M	M_0_: 26	M_0_: 13

The staging reported here is the histological staging obtained after surgical resection

**Table 2:** Primer sequences designed for RT–PCR studies

**ADAM (accession number)**	**Tm**	**Cycles**	**Primer**	**Sequence**
ADAM-8 (NM_001109)	56	38	Antisens	5′TTCTTGCTGTGGTCCTGGTTCA3′
			Sens	5′GTGAATCACGTGGACAAGCTAT3′
ADAM-9 (U41766)	60	28	Antisens	5′TTTTCCCGCCACTGCACGAAGT3′
			Sens	5′AGAAGAGCTGTCTTGCCACAGA3′
ADAM-10 (AF009615)	60	28	Antisens	5′GGTTGGCCAGATTCAACAAAAC3′
			Sens	5′TTTGGATCCCCACATGATTCTG 3′
ADAM-12 (AF023476)	56	35	Antisens	5′TTCCTGCTGCAACTGCTGAACA3′
			Sens	5′GGAATTGTCATGGACCATTCAG3′
12 spliced long (NM_003474)	58	36	Antisens	5′TTGAGGGGTCTGCTGATGTCAA3′
			Sens	5′TTGGCTTTGGAGGAAGCACAGA3′
12 spliced short (NM_021641)	58	40	Antisens	5′GCAAAGCCACAGAGTCAATGCT3′
			Sens	5′ TTGGCTTTGGAGGAAGCACAGA3′
ADAM-15 (BC014566)	60	28	Antisens	5′TTCGAAGAGGCAGCTGCCCATT3′
			Sens	5′AACATGGACCACTCCACCAGCA3′
ADAM-17 (U69611)	60	28	Antisens	5′TTCATCCACCCTCGAGTTCCCA3′
			Sens	5′TACAAAGGAAGCTGACCTGGTT3′
ADAMTS-1 (AF207664)	60	28	Antisens	5′TTCACTTCGATGTTGGTGGCTC3′
			Sens	5′CAGCCCAAGGTTGTAGATGGTA3′
ADAMTS-2 (NM_014244)	66	32	Antisens	5′GGCTGCAGCGGGACCAGTGGAA3′
			Sens	5′GAACCATGAGGACGGCTTCTCCT3′
ADAMTS-12 (AJ250725)	62	35	Antisens	5′AAGTTGTGCCTCTCCCACTTCT3′
			Sens	5′CTGCCATGGACTGACTGGATTT3′

**Table 3:** Expression pattern of several ADAMs and ADAMTS in non-small-cell lung carcinomas measured by semiquantitative RT–PCR

	**Tumours**	**Controls**
ADAM-8	0.06±0.006	0.05±0.01
ADAM-9	0.6±0.1	0.3±0.07
ADAM-10	0.7±0.14	0.34±0.07
ADAM-12	0.3±0.09^*^	0.05±0.004
ADAM-15	0.8±0.11	0.46±0.07
ADAM-17	0.2±0.07	0.16±0.03
ADAMTS-1	0.19±0.05^*^	0.3±0.06
ADAMTS-2	0.31±0.07	0.37±0.11
ADAMTS-12	0.3±0.08	0.23±0.05

Results are expressed as arbitrary units (AU) (mean±s.e.m.) and are normalised for 28S rRNA expression.

^*^=*P*<0.05 *vs* controls.
